# Three-Dimensional Evaluation of Isodose Radiation Volumes in Cases of Severe Mandibular Osteoradionecrosis for the Prediction of Recurrence after Segmental Resection

**DOI:** 10.3390/jpm12050834

**Published:** 2022-05-20

**Authors:** Haye H. Glas, Joep Kraeima, Silke Tribius, Frank K. J. Leusink, Carsten Rendenbach, Max Heiland, Carmen Stromberger, Ashkan Rashad, Clifton D. Fuller, Abdallah S. R. Mohamed, Stephen Y. Lai, Max J. H. Witjes

**Affiliations:** 1 Department of Oral and Maxillofacial Surgery, University Medical Center Groningen, University of Groningen, 9713GZ Groningen, The Netherlands; j.kraeima@umcg.nl (J.K.); m.j.h.witjes@umcg.nl (M.J.H.W.); 2 Hermann-Holthusen-Institute for Radiation Oncology, Asklepios Hospital St. Georg, University Medical Center Hamburg-Eppendorf, 20246 Hamburg, Germany; s.tribius@asklepios.com; 3 Department of Oral and Maxillofacial Surgery, Amsterdam University Medical Center, 1100DD Amsterdam, The Netherlands; f.leusink@amsterdamumc.nl; 4 Department of Oral and Maxillofacial Surgery, Charité–Universitätsmedizin, Corporate Member of Freie Universität Berlin, Humboldt-Universität zu Berlin and Berlin Institute of Health, 12203 Berlin, Germany; carsten.rendenbach@charite.de (C.R.); max.heiland@charite.de (M.H.); 5 Department of Radiation Oncology and Radiation Therapy, Charité–Universitätsmedizin, Corporate Member of Freie Universität Berlin, Humboldt-Universität zu Berlin and Berlin Institute of Health, 12203 Berlin, Germany; carmen.stromberger@charite.de; 6 Department of Oral, Maxillofacial and Facial Plastic Surgery, RWTH Aachen University Hospital, 52074 Aachen, Germany; arashad@ukaachen.de; 7 Department of Radiation Oncology, Division of Radiation Oncology, The University of Texas MD Anderson Cancer Center, Houston, TX 77030, USA; cdfuller@mdanderson.org (C.D.F.); asmohamed@mdanderson.org (A.S.R.M.); sylai@mdanderson.org (S.Y.L.); 8 Department of Head and Neck Surgery, Division of Surgery, The University of Texas MD Anderson Cancer Center, Houston, TX 77030, USA

**Keywords:** osteoradionecrosis, mandible, radiotherapy, surgery, computer assisted surgery, virtual surgical planning

## Abstract

Background: Pre-operative margin planning for the segmental resection of affected bone in mandibular osteoradionecrosis (ORN) is difficult. The aim of this study was to identify a possible relation between the received RT dose, exposed bone volume and the progression of ORN after segmental mandibular resection. Method: Patients diagnosed with grade 3-4 ORN for which a segmental resection was performed were included in the study. Three-dimensional reconstructions of RT isodose volumes were fused with postoperative imaging. The primary outcome was the recurrence of ORN after segmental resection. Subsequently, RT exposed mandibular bone volumes were calculated and the location of the bone cuts relative to the isodose volumes were assessed. Results: Five out of thirty-three patients developed recurrent ORN after segmental mandibular resection. All cases with recurrent ORN were resected inside an isodose volume of ≥56 Gy. The absolute mandibular volume radiated with 56 Gy was significantly smaller in the recurrent group (10.9 mL vs. 30.7 mL, *p* = 0.006), as was the proportion of the mandible radiated with 56 Gy (23% vs. 45%, *p* = 0.013). Conclusion: The volume of radiated bone was not predictive for risk of progression. The finding that recurrent ORN occurred with bone resection margins within the 56 Gy isodose volume suggests that this could serve as a starting point for the pre-operative planning of reducing the risk of ORN recurrence.

## 1. Introduction

Osteoradionecrosis (ORN) of the mandible is a late complication of radiotherapy (RT). ORN most commonly occurs in the tooth-bearing body of the mandible [1]. It is described as exposed irradiated bone that fails to heal over a period of three months without evidence of a persisting or recurrent tumour [2]. Incidence of ORN is reported to occur in 1–15% of head and neck cancer patients with a median latency of 1 to 2 years after RT [1,3,4,5,6,7]. Factors such as the received RT-dose, the volume of mandible included in the planning target volume (PTV) as well as the fractionation schedule are known to influence the occurrence of ORN [3,4,6,8,9]. Other risk factors associated with the development of ORN are surgery to the mandible, dental condition and pre- or post-RT tooth extractions, as well as continued smoking [1,5,10,11,12,13]. There is no association between concomitant chemotherapy and the incidence of ORN [12]. The initiation of ORN is mainly reported after traumatic events to the bone but is also known to occur spontaneously [14,15].

Mental neuropathy, dehiscent bone or fistulas may be predictors for ORN as well as medication-related osteonecrosis of the jaw (MRONJ); however, ORN patients demonstrate significantly more pathological fractures, skin fistulae and pain compared to MRONJ [16,17]. Despite some similarities, ORN and MRONJ are considered two distinct pathological entities [16].

The treatment of patients with ORN depends on the extent of the affected bone and may consist of antibiotics, debridement, sequestrectomy, hyperbaric oxygen therapy, or a combination of the aforementioned techniques [18]. Treatment of severe mandibular ORN often requires the surgical removal of the affected bone and segmental resection is often necessary. ORN of the jaw is often classified into several stages that describe the severity or progression of the disease. The classification used by Marx et al. defines three stages of ORN, in which the third stage includes pathological fractures, orocutaneous fistulas or radiographic evidence of resorption of the inferior border.

The risk of developing ORN is associated with a dose of >60 Gy to the bone [8,19,20], or a mean dose to the total mandibular volume of >48 Gy [21]. Additionally, a gross tumour volume (GTV) dose of >54 Gy is related to an increased risk of developing ORN [4]. Furthermore, the risk of developing ORN is also reported to be related to the volume of bone and the received RT dose. Emami et al. reported a 5% risk of developing ORN within the 5 years following RT when 2/3 of the mandible is radiated with more than 60 Gy, which is equal to more than 65 Gy when approximately 1/3 of the mandible is exposed [9]. Tsai et al. reported a matched case–control analysis, with a significant difference between the volume of the mandible in the two groups receiving doses between 50 Gy (V_50_) and 60 Gy (V_60_) [6]. Abdallah et al. reported on a case–control matched study with significant higher dose-volume histogram () bins from V35 to v73 in the ORN cohort [21]. A DVH is used to relate radiation dose to tissue volume. It can be concluded that the risk of ORN increases with radiation dose and radiated mandibular volume, with an incremental increasing risk for ORN at doses of above 50 Gy.

Currently, the position of bone cuts for mandibular segmental resection are based on the clinical inspection of the lesion and pre-operative imaging such as panoramic X-rays or (CB)CT/MRI. The use of Technetium-bone scans has been described as a method to identify the affected bone [22]. Using DCE-MRI, differences in vascular leakiness can be measured between affected and healthy bone tissue [23]. However, surgeons often struggle with the decision of where to make cuts in the mandible and the resulting margins. The most commonly used technique is to remove bone until healthy, bleeding bone is visible [24]. Others have described using tetracycline as a fluorescent marker to discriminate between vital and necrotic bone [25,26,27]. As was described by Kraeima et al., the 3D- visualization of the isodose lines obtained using RT planned with IMRT in relation to the mandibular bone can support preoperative planning [28]. This method provides a potential decision-making tool that can be used pre-operatively. However, sufficient data on the relationship between the received RT dose and the ideal location for the bone cuts when a segmental mandibular resection is performed are not available. Defining this relationship is important in order to determine a cut-off dose that may possibly be used for the pre-operative planning of the surgical resection or placement of screws for fixating osteosynthesis materials.

This study describes a retrospective analysis of an international multi-institutional database for patients with severe ORN that required surgical treatment. The aim of the study was to identify the relationship between the received RT dose, exposed bone volume and progression of ORN after segmental mandibular resection in order to support the preoperative planning of the bone cuts.

## 2. Materials and Method

### 2.1. Patients

An international consortium of medical centres collected retrospective data on patients who underwent segmental mandibular resection as treatment for ORN. The selection focused on patients who developed severe (Marx classification grade 3) ORN after RT/chemoRT and were treated in the following centres: University of Texas MD Anderson Cancer Center (Houston, TX, USA), Charité-Universitätsmedizin Berlin (Berlin, Germany), University Medical Center Hamburg-Eppendorf (Hamburg, Germany), Amsterdam University Medical Center (Amsterdam, the Netherlands) and the University Medical Center Groningen (Groningen, the Netherlands). Written informed consent was obtained from all subjects involved in the study. The study was approved by the ethical committee (Berlin EA1/206/18).

Inclusion criteria were as follows: (1) Patients diagnosed with Marx grade 3 ORN of the mandible after IMRT for which a segmental resection was performed; (2) patients who underwent IMRT with curative intent as part of their initial treatment after the confirmed pathological diagnosis of oral or oropharyngeal squamous cell carcinoma; (3) availability of the following data: radiotherapy-CT scan and radiation plan (DICOM-RT) to reconstruct 3D-isodose fields and the postoperative imaging data to derive the performed resection, either by CT scan or orthopantomogram (OPT). Furthermore, patients who received brachytherapy or previous head and neck RT were excluded.

The following patient characteristics were recorded: age, gender, smoking and alcohol consumption, tumour stage and location, primary treatment (surgery, RT, chemoradiation or a combination of aforementioned), months between RT and diagnosis of ORN, dental status, HBO therapy, RT dose and fractionation schedule. The dental status was retrieved from clinical files, including performed extractions or the invasive treatment of any other conditions. If such data were not available, patients were marked edentulous when the RT planning CT did not reveal any elements.

### 2.2. Processing of Imaging Data

**Figure 1:**
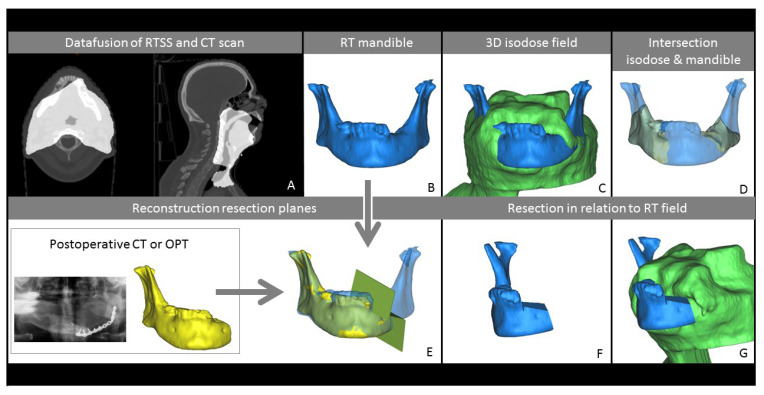
Describing the workflow of data fusion and segmentation, including reconstruction of the performed segmental resection. (**A**). Data fusion of radiotherapy planning files (RTSS) and RT CT scan. (**B**). 3D model of the mandible from RT CT scan. (**C**). In green, the 3D reconstruction of the 56 Gy isodose volume. (**D**). In yellow, the volume of the mandible radiated with 56 Gy (Vm56). (**E**). Reconstruction of performed segmental resection using either a postoperative CT or OPT. (**F**). Mandible after segmental resection. (**G**). Mandible after segmental resection in relation to the 56 Gy isodose volume (V56).

For every case, the RT planning CT scan was selected for the 3D-segmentation of the bone (e.g., the mandible). The DICOM-RT clinical treatment plans were uploaded to the RT planning software research database (Mirada, Mirada Medical, Oxford, UK) and fused with the selected CT dataset. The 56 Gy and PTV isodose curves were visualized and exported as RTSS files. Subsequently, these RTSS files were fused with the RT planning CT using a similar conversion method to that described by Kraeima et al. [29], using Matlab 2018a (MathWorks, Natick, MA, USA). After data fusion, a 3D-virtual model of both the involved bone, 56 Gy and PTV isodose volumes were produced using ProPlan CMF 3.0 (Materialise, Leuven, Belgium). Figure 1 presents a stepwise overview of the workflow.

Postoperative imaging was used to derive the margins of the performed segmental resection, using either a CT scan or OPT. When a postoperative CT scan was available, 3D-segmentation of the resected mandible was performed and registered with the 3D RT reconstruction. When the shape of the mandible significantly changed due to the reconstruction, this registration was performed twice, once for each segment. Hereafter, cutting planes superimposing the performed resection onto the RT bone model were constructed. In case an OPT was used, resection planes were translated manually using screen-to-screen comparison onto the RT bone model.

### 2.3. Measurements

**Figure 2:**
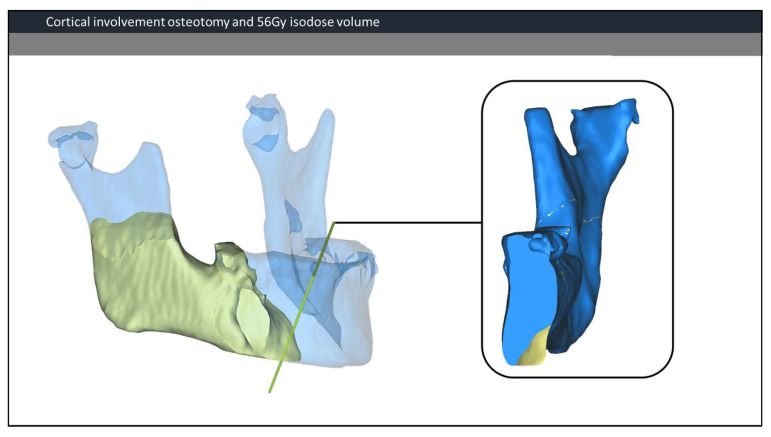
Cortical involvement osteotomy and 56 Gy isodose volume. On the left, an overview of the mandible and the 56 Gy isodose volume (yellow). The osteotomy plane is visualized in green. On the right side, a view perpendicular to the osteotomy plane. In this case, only the lingual cortex is involved.

Volume measurements were performed on the combined dataset, including total mandible volume (Vm), 56 Gy and PTV isodose volumes (V56, V-PTV), volume of mandible inside the 56 Gy (Vm56) and PTV (Vm-PTV) isodose volume. The PTV resembled the high-dose volume and included the gross tumour volume (GTV) and the clinical target volume with an additional set-up margin. The PTV dose is typically equal or higher than 56 Gy. Further measurements included volume of the resection of the mandible (VmR), and residual volume of V56 and V-PTV after resection surgery (Vm56R, Vm-PTV-R). Besides absolute volumes, the distribution of the volumes was calculated as a percentage of total mandibular volume. Furthermore, for each resection, we assessed whether the resection was performed inside the Vm56 and Vm-PTV volume. If the resection was performed inside the Vm56 volume, the involvement of the lingual and/or buccal cortex was noted. Figure 2 illustrates an example of lingual involvement in the osteotomy with the Vm56. Moreover, the progression of ORN after segmental resection was used as an outcome measure. A Kolmogorov–Smirnov analysis for normal distribution was performed. A student’s *t*-test for normally distributed data and the Mann–Whitney U (MWW) test for skewed data were used to detect significant differences between recurrent and non-recurrent cases.

## 3. Results

### 3.1. Patient Characteristics

A total of 33 patients who underwent segmental mandibulectomy for severe ORN following RT/chemoRT were included in the study. Patients were treated for primary ORN between 2008 and 2018. Follow-up after initial ORN surgery was 69 months (range 19–142 months). Five patients were diagnosed with recurrence of ORN after initial segmental resection. A complete list of all patient characteristics can be found in Table 1.

A total of 75 patients diagnosed with severe ORN were assessed. Forty-two patients were excluded from the analysis. Reasons for exclusion included no segmental resection (12), incomplete RT planning data or unavailable data for reconstruction (13), incomplete postoperative imaging data (11), prior RT (3), additional brachytherapy (1), total radiation dose <56 Gy (1) and unavailable patient record (1).

### 3.2. Recurrent Cases

**Figure 3:**
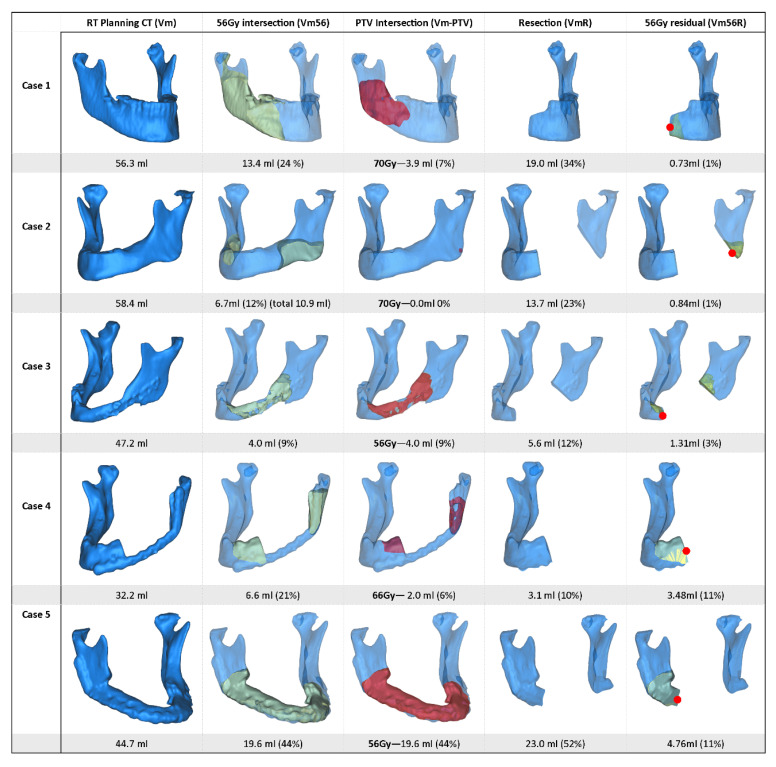
Overview of patients with recurrence of ORN after segmental resection. The first column illustrates the 3D segmentation of the mandible during radiotherapy, representing the total mandibular volume (Vm). The second column illustrates, in yellow, the volume of the mandible inside the 56 Gy isodose (Vm56). The third column, in red, represents the volume of the mandible inside the PTV (Vm-PTV). The forth column shows the postoperative situation after segmental resection (VmR). The last column shows the residual volume of Vm56 after resection surgery (Vm56R). The red dots in the last column indicate the location of the ORN recurrence.

A total of five patients were diagnosed with recurrent ORN after mandibular segmental resection, with initial diagnoses of ORN occurring 16.2 months (range 1–34) after RT. The median age of patients was 60 years (range 53–66 years). The mean radiation dose was 64 Gy (SD 6.4 Gy). Figure 3 illustrates the recurrent ORN cases, including the radiated mandibular volume, performed resection and location of the recurrent ORN. Four patients were edentulous at the time of RT and three patients presented osteosynthesis material in situ during RT.

### 3.3. Measurements

The mean mandible volume was 62.7 mL (range 26.4–95mL). On average, 42% (range 9–83%) of the mandible was radiated with at least 56 Gy (Vm56/Vm), and 15% (range 0–51%) of the mandible received the PTV dose, which ranged from 56 Gy to 72 Gy. On average 35% (range 7–78%) of the mandible was resected. The total mandibular volume of the recurrent ORN group was smaller compared to the non-recurrent group (65.4 mL vs. 47.8 mL, *t*-test *p* = 0.045). Additionally, Vm56 volume was significantly smaller in the recurrent group (10.9 mL vs. 30.7ml, *t*-test *p* = 0.006). The proportion of mandible radiated with 56 Gy was smaller for the recurrent group than for the non-recurrent group (23% vs. 45% (MWW *p* = 0.013)). Resections performed in the non-recurrent group included a larger proportion (37%) of the total mandibular volume compared to the recurrent group (26%), although this was not significant (MWW *p* = 0.268). Two recurrences of ORN in patients occurred for those who received the highest doses of RT of 70 Gy or more while 20 patients showed no recurrences. The overall volume measures were smaller in recurrent cases than in non-recurrent cases. A complete overview of the volume measurements and volume distribution is provided in Table 2.

A total of 60 osteotomy planes were reconstructed (8 recurrent cases, 52 non-recurrent cases). In total, 14 resections were outside the Vm56. Thus, the margin was in bone that received a lower RT dose. Of the 46 resections inside the Vm56, 10 planes intersected the Vm56 at the lingual cortex only. The remaining 36 resections intersected the Vm56 in both the lingual and buccal cortex. Of the eight osteotomies in the recurrent ORN group, five intersected the Vm56 bicortically, one on the lingual side and the remaining two were performed outside the Vm56, thus in bone with a dose of less than 56 Gy.

## 4. Discussion

In the present study, five out of thirty-three patients developed a recurrence of ORN after segmental resection. Two of the five patients received a maximum RT dose of 56 Gy and for the other three patients the ORN recurred in a mandibular volume that was exposed to more than 56 Gy RT. No recurrence was observed with margins placed in the mandibular volume exposed to less than 56 Gy. Although not significantly different, the resection volumes in the non-recurrent ORN group were larger than in the recurrent ORN group, possibly indicating that sufficiently large resection volumes of radiated bone may reduce the chance of recurrence. This study is a first attempt to involve radiation dose in surgical decision making in the treatment of ORN. Because of the retrospective nature of the study, the data were focused on what could be reliably extracted, the placement of the bone cut, the isodose and the progression of ORN.

Aiming to place the bone margin outside the 56 Gy volume may reduce the risk of ORN recurrence. Although this concept is supported by general findings in the literature that the risk of ORN increases incrementally with doses of more than 50 Gy, the data from the current cohort do not support this [4,6,8,19,21]. Moreover, it has been suggested that the risk of recurrent ORN after surgical resection is associated with multiple factors and should most likely be considered as such [1,3,4,5,6,7,8,9,10,11,13]. Thus, the approach of making resection-margin decisions based on isodose distribution needs to be approached with caution. From this data, the concept of always placing the bone margins outside the 56 Gy isodose volume could not be applied uniformly.

Studer at al. reported about 42 patients for whom a mean mandible volume of 4.6% received the prescribed dose (71 Gy) [7]. Compared to our cohort of patients who received a prescribed dose of a minimum of 70 Gy, we found 10% for the non-recurrent ORN group and 3% for the recurrent ORN group. According to Emami et al., the risk of developing an ORN is 5% in 5 years if one third of the mandible is exposed to 65 Gy, or if two thirds is exposed to 60 Gy [9]. In our study, on average, 42% of the mandible received a dose of 56 Gy. However, for the recurrent cases this proportion was lower (23%) than for the non-recurrent ORN cases (45%). This is also lower than the DVH constraints of V58 < 25% proposed by Abdallah et al. [21].

Mandibular surgery increases the risk of the development of ORN [4], where marginal or periosteal bone resection impose the highest risk, followed by segmental or no resection [19]. In the recurrent ORN group, three of the five patients had osteosynthesis material in place during RT/chemoRT. In two of these patients, a segmental resection and reconstruction was performed during primary resection. One patient underwent reconstruction with a fibula graft, the other solely with osteosynthesis. The third case had a mandibular fracture that was sustained during radical surgery, for which internal fixation was indicated. In the non-recurrent ORN group, only four of the twenty-eight patients had already undergone reconstructive surgery. This might also explain the difference in the total mandibular bone volume at the time of RT between the recurrent and non-recurrent ORN group. The resection in recurrent case 4 consisted of removing the condyle and osteosyntheses material, and there was no bone cutting. However, this case is considered as a bicortical involvement of the osteotomy at the Vm56 volume, because the Vm56 was not completely removed during surgery. For recurrent case 5, one of the osteotomies was performed in the fibula graft and not in the mandibular bone. For the same reason mentioned before, this osteotomy was also considered as bicortical involvement.

In this study, a non-systematic analysis of retrospective data on ORN patients was conducted to establish evidence to support potential dose–volume criteria for pre-operative decision making for 3D-virtual surgical planning. Data from 33 patients were used from an initial cohort of 75 patients (incomplete records in 42 cases), emphasizing the need for a multicentre approach. Despite the multicentre approach, only a limited number of patients could be included. The limited data availability and the resulting statistical power is the main limitation of this study. Moreover, as treatment and surgical reconstruction techniques differ between patients, but also between surgeons and health centres, comparing individual patients is even more challenging. In addition, ORN is a multifactorial disorder, and due to the retrospective nature of the study, not all factors could be included. We did not find a relation between the proportion of recurrences and the RT dose or dose-volumes. Perhaps the group was too small to draw conclusions related to RT dose and dose volumes. This study was not set up as an investigation of the causes of ORN, but for the risk of recurrence. Therefore, the data retrieved from these cases should not be viewed similarly to those in previous reports on the relationship of RT dose, dose volumes and the risk of developing ORN. The unexpected finding that mandibular RT dose volumes were smaller in the recurrent ORN group is perhaps more a consequence of all patients already having ORN and less related to recurrence.

The surgical technique and choices regarding the placement of the bone cuts and reconstruction are relevant for the risk of the progression of ORN. The risk of ORN progression is not solely based on the RT dose given to the bone. Other factors related to surgery, such as vascularization of the remaining bone, quality of the covering soft tissue as well as patient-related factors, including smoking and health status. Placing the bone margin in the isodose volume with the lowest risk of the recurrence of ORN may be just one factor in the process. The importance of planning the bone margin is even more essential for 3D-surgical planning of the bone reconstruction. The traditional surgical approach would be free-hand resection and shaping of the composite flap in the OR without 3D-virtual planning. In the OR, regardless of the decision to utilize 3D-planning, the surgeon is faced with the problem of where to cut and the following question remains: ‘is bleeding bone a safe criterion?’.

## 5. Conclusions

All of the patients who experienced the progression of ORN after the surgical removal of the affected mandibular bone were resected inside the 56 Gy volume. Although the volume measurements alone are not predictive for progression, the authors suggest that the use of 3D-isodose volumes may be an option to avoid areas at risk for ORN during one-stage resection and reconstructive surgery. This approach warrants further evaluation.

## Figures and Tables

**Table 1:** Patient characteristics.

		Value	%
Age			
	Median (range)	60	(43–76)
sex			
	male	21	64%
	Female	12	36%
Smoking status			
	Never	6	23%
	Former	11	42%
	Current	9	35%
	unknown	7	
Smoking pack-year			
	Mean (SD)	31	(23)
Alcohol history			
	occasional	5	19%
	Former	11	41%
	Current	13	48%
	unknown	6	
Tumour location			
	Base of tonque	11	48%
	Tonsil	8	35%
	Other	4	17%
	unknown	10	
T stage			
	T1	1	3%
	T2	12	38%
	T3	6	19%
	T4	13	41%
	unknown	1	
N stage			
	N0	6	19%
	N1	5	16%
	N2	21	66%
	unknown	1	
Primary treatment			
	RT	3	9%
	Surgery + RT	7	21%
	RCT	20	61%
	Surgery + RCT	3	9%
Time RT-ORN			
	Months (range)	28	(1–76)
Reconstruction method			
	Fibula (unknown)	12	
		21	(21)
Follow-up initial ORN			
	Months (range)	69	(19–142)
Dental status			
	Edentulous	11	33%
	dental extractions	16	59%
	unknown	6	
HBO therapy		18	55%
Radiation dose			
	Median (range)	70 Gy	(56–72)
Radiation fractions			
	Median (range)	33	(28–45)
	RT = radiotherapy		
	RCT = radiotherapy + chemotherapy		

**Table 2:** Mean, minimum and maximum volumes of mandible and RT isodose fields. Volume of mandible (Vm), 56 Gy and PTV isodose volume (V56, V-PTV). Volume mandible inside 56 Gy isodose and PTV (Vm56, Vm-PTV). Volume of resection (VmR) and residual volume of Vm56 and Vm-PTV after resection (Vm56R, Vm-PTV-R). Significant differences between recurrent and non-recurrent group are highlighted in red.

		Vm	V56	V-PTV	Vm56	Vm-PTV	Vm56R	Vm-PTV-R	VmR	Vm56/Vm	Vm-PTV/Vm	VmR/Vm
**Total (n = 33)**	**Mean (mL)**	**62.7**	**772.8**	**221.5**	**27.7**	**9.2**	**9.6**	**3.3**	**39.4**	**42%**	**15%**	**35%**
	min	26.4	33.8	0.0	4.0	0.0	0.0	0.0	23.9	9%	0%	7%
	max	95.0	1760.9	623.2	77.5	38.6	53.1	31.4	36.0	83%	51%	78%
**Non-recurrent (n = 28)**	**Mean (mL)**	**65.4**	**843.5**	**238.6**	**30.7**	**9.8**	**10.9**	**3.8**	**40.3**	**45%**	**15%**	**37%**
	min	26.4	183.6	0.0	4.5	0.0	0.0	0.0	23.9	10%	0%	7%
	max	95.0	1760.9	623.2	77.5	38.6	53.1	31.4	36.0	83%	51%	78%
**Recurrent (n = 5)**	**Mean (mL)**	**47.8**	**376.9**	**125.7**	**10.9**	**5.9**	**2.2**	**0.0**	**34.8**	**23%**	**13%**	**26%**
	min	32.2	33.8	33.8	4.0	0.0	0.7	0.0	29.1	9%	0%	10%
	max	58.4	763.0	212.1	19.6	19.6	4.8	0.0	35.1	44%	44%	52%
**>70 Gy Non-recurrent (n = 20)**	**Mean (mL)**	**67.8**	**950.7**	**235.6**	**29.9**	**6.7**	**7.7**	**1.5**	**43.7**	**43%**	**10%**	**34%**
	min	26.4	183.6	0.0	6.7	0.0	0.0	0.0	23.9	12%	0%	7%
	max	95.0	1760.9	604.8	77.5	33.7	32.5	10.2	42.2	83%	44%	63%
**>70 Gy Recurrent (n = 2)**	**Mean (mL)**	**57.3**	**751.3**	**196.7**	**12.2**	**1.9**	**0.8**	**0.0**	**41.0**	**21%**	**3%**	**29%**
	min	56.3	739.7	181.3	10.9	0.0	0.7	0.0	42.6	19%	0%	23%
	max	58.4	763.0	212.1	13.4	3.9	0.8	0.0	39.4	24%	7%	34%

## Data Availability

The data that support the findings of this study are available from the corresponding author, H.H.G, upon reasonable request.

## Abbreviations

3D	Three-dimensional
GTV	Gross tumour volume
DVH	Dose volume histogram
IMRT	Intensity modulated radiotherapy
MRONJ	Medication-related osteonecrosis of the jaw
ORN	Osteoradionecrosis
PTV	Planning target volume
RT	Radiotherapy
Vm	Mandible volume
VSP	Virtual surgical planning

## References

[1] Reuther T., Schuster T., Mende U., Kübler A. (2003). Osteoradionecrosis of the jaws as a side effect of radiotherapy of head and neck tumour patients—A report of a thirty year retrospective review. Int. J. Oral Maxillofac. Surg..

[2] Mallya S.M., Tetradis S. (2018). Imaging of Radiation- and Medication-Related Osteonecrosis. Radiol. Clin. N. Am..

[3] Mendenhall W.M., Suárez C., Genden E.M., De Bree R., Strojan P., Langendijk J.A., Mäkitie A.A., Smee R., Eisbruch A., Lee A.W. (2018). Parameters Associated With Mandibular Osteoradionecrosis. Am. J. Clin. Oncol. Cancer Clin. Trials.

[4] Lee I.J., Koom W.S., Lee C.G., Kim Y.B., Yoo S.W., Keum K.C., Kim G.E., Choi E.C., Cha I. (2009). Risk Factors and Dose–Effect Relationship for Mandibular Osteoradionecrosis in Oral and Oropharyngeal Cancer Patients. Int. J. Radiat. Oncol. Biol. Phys..

[5] Pereira I., Firmino R., Meira H., Vasconcelos B., Noronha V., Santos V. (2018). Osteoradionecrosis prevalence and associated factors: A ten years retrospective study. Med. Oral Patol. Oral y Cir. Bucal.

[6] Tsai C.J., Hofstede T.M., Sturgis E.M., Garden A.S., Lindberg M.E., Wei Q., Tucker S.L., Dong L. (2012). Osteoradionecrosis and Radiation Dose to the Mandible in Patients With Oropharyngeal Cancer. Int. J. Radiat. Oncol. Biol. Phys..

[7] Studer G., Studer S.P., Zwahlen R.A., Huguenin P., Grätz K.W., Lütolf U.M., Glanzmann C. (2006). Osteoradionecrosis of the mandible: Minimized risk profile following intensity-modulated radiation therapy (IMRT). Strahlenther. Onkol..

[8] Murray C.G., Herson J., Daly T.E., Zimmerman S. (1980). Radiation necrosis of the mandible: A 10 year study. Part I. Factors influencing the onset of necrosis. Int. J. Radiat. Oncol. Biol. Phys..

[9] Emami B. (1991). Tolerance of Normal Tissue to Irradiation. Int. J. Radiat. Oncol. Biol. Phys..

[10] Aarup-Kristensen S., Hansen C.R., Forner L., Brink C., Eriksen J.G., Johansen J. (2019). Osteoradionecrosis of the mandible after radiotherapy for head and neck cancer: Risk factors and dose-volume correlations. Acta Oncol..

[11] Manzano B.R., Santaella N.G., Oliveira M.A., Rubira C.M.F., de Santos P.S. (2019). Retrospective study of osteoradi-onecrosis in the jaws of patients with head and neck cancer. J. Korean Assoc. Oral Maxillofac. Surg..

[12] Studer G., Grätz K.W., Glanzmann C. (2004). Osteoradionecrosis of the Mandibula in Patients Treated with Different Fractionations. Strahlenther. Onkol..

[13] Glanzmann C., Grätz K. (1995). Radionecrosis of the mandibula: A retrospective analysis of the incidence and risk factors. Radiother. Oncol..

[14] Marx R.E. (1983). A New Concept of Its Pathophysiology. Growth.

[15] Wanifuchi S., Akashi M., Ejima Y., Shinomiya H., Minamikawa T., Furudoi S., Otsuki N., Sasaki R., Nibu K.-I., Komori T. (2016). Cause and occurrence timing of osteoradionecrosis of the jaw: A retrospective study focusing on prophylactic tooth extraction. Oral Maxillofac. Surg..

[16] Grisar K., Schol M., Schoenaers J., Dormaar T., Coropciuc R., Poorten V.V., Politis C. (2016). Osteoradionecrosis and medication-related osteonecrosis of the jaw: Similarities and differences. Int. J. Oral Maxillofac. Surg..

[17] Fortunato L., Amato M., Simeone M., Bennardo F., Barone S., Giudice A. (2018). Numb chin syndrome: A reflection of malignancy or a harbinger of MRONJ? A multicenter experience. J. Stomatol. Oral Maxillofac. Surg..

[18] Nadella K.R., Kodali R.M., Guttikonda L.K., Jonnalagadda A. (2015). Osteoradionecrosis of the Jaws: Clinico-Therapeutic Management: A Literature Review and Update. J. Maxillofac. Oral Surg..

[19] Studer G., Bredell M., Studer S., Huber G., Glanzmann C. (2016). Risikoprofil für Osteoradionekrosen des Kiefers in der IMRT-Ära. Strahlenther. Onkol..

[20] Ben-David M.A., Diamante M., Radawski J.D., Vineberg K.A., Stroup C., Murdoch-Kinch C.-A., Zwetchkenbaum S.R., Eisbruch A. (2007). Lack of Osteoradionecrosis of the Mandible After Intensity-Modulated Radiotherapy for Head and Neck Cancer: Likely Contributions of Both Dental Care and Improved Dose Distributions. Int. J. Radiat. Oncol..

[21] Anderson Head and Neck Cancer Symptom Working Group (2017). Dose-volume correlates of mandibular osteoradionecrosis in Oropharynx cancer patients receiv-ing intensity-modulated radiotherapy: Results from a case-matched comparison. Radiother. Oncol..

[22] Dore F., Filippi L., Biasotto M., Chiandussi S., Cavalli F., di Lenarda R. (2009). Bone scintigraphy and SPECT/CT of bisphos-phonate-induced osteonecrosis of the jaw. J. Nucl. Med..

[23] Mohamed A.S., He R., Ding Y., Wang J., Fahim J., Elgohari B., Elhalawani H., Kim A.D., Ahmed H., Garcia J.A. (2020). Quantitative Dynamic Contrast-Enhanced MRI Identifies Radiation-Induced Vascular Damage in Patients With Advanced Osteoradionecrosis: Results of a Prospective Study. Int. J. Radiat. Oncol..

[24] Zaghi S., Miller M., Blackwell K., Palla B., Lai C., Nabili V. (2012). Analysis of surgical margins in cases of mandibular oste-oradionecrosis that progress despite extensive mandible resection and free tissue transfer. Am. J. Otolaryngol..

[25] Alam D.S., Nuara M., Christian J. (2009). Analysis of Outcomes of Vascularized Flap Reconstruction in Patients with Advanced Mandibular Osteoradionecrosis. Otolaryngol. Neck Surg..

[26] Curi M.M., dos Santos M.O., Feher O., Faria J.C.M., Rodrigues M.L., Kowalski L.P. (2007). Management of Extensive Osteoradionecrosis of the Mandible With Radical Resection and Immediate Microvascular Reconstruction. J. Oral Maxillofac. Surg..

[27] Marx R.E. (1983). A new concept in the treatment of osteoradionecrosis. J. Oral Maxillofac. Surg..

[28] Kraeima J., Steenbakkers R.J.H.M., Spijkervet F.K.L., Roodenburg J.L.N., Witjes M.J.H. (2018). Secondary surgical man-agement of osteoradionecrosis using three-dimensional isodose curve visualization: A report of three cases. Int. J. Oral Maxillofac. Surg..

[29] Kraeima J., Schepers R.H., van Ooijen P.M.A., Steenbakkers R.J.H.M., Roodenburg J.L.N., Witjes M.J.H. (2015). Integration of oncologic margins in three-dimensional virtual planning for head and neck surgery, including a validation of the software pathway. J. Cranio Maxillofac. Surg..

